# Evaluation of Anchote (*Coccinia abyssinica*) Genotypes and Processing Methods for Nutritional Compositions

**DOI:** 10.1002/fsn3.71288

**Published:** 2025-11-28

**Authors:** Bogalech Negassa, Amsalu Nebiyu, Weyessa Garedew, Lord Abbey, Raphael Ofoe, Tessema Astatkie, Chala Gowe Kuyu

**Affiliations:** ^1^ Department of Horticulture and Plant Sciences, College of Agriculture and Veterinary Medicine Jimma University Jimma Ethiopia; ^2^ Department of Plant, Food, and Environmental Sciences, Faculty of Agriculture Dalhousie University Bible Hill Nova Scotia Canada; ^3^ Department of Engineering, Faculty of Agriculture Dalhousie University Bible Hill Nova Scotia Canada; ^4^ Department of Postharvest Management, College of Agriculture and Veterinary Medicine Jimma University Jimma Ethiopia

**Keywords:** anchote, cooking method, proximate composition

## Abstract

Anchote, a versatile root crop deeply ingrained in Ethiopian culture, plays a vital role in food, medicine, social gatherings, and the economy. Most predominant in Ethiopia's southern, and southwestern regions, anchote stands out as a high‐yield crop with superior biochemical characteristics compared to other root vegetables. Despite its potential, the cultivation of anchote remains localized, prompting on‐farm research to explore the nutritional content of different anchote genotypes and to find optimal cooking methods to preserve these essential elements. This study evaluated the proximate composition of three anchote genotypes, two local landraces (LV1, LV2) and an improved variety (Desta 01), under two cooking methods (boiled peeled vs. unpeeled roots) in a factorial arrangement on‐farm trial across four sites. The results indicated no significant differences (*p* > 0.05) in most proximate compositions among genotypes and treatments, with the exception of ash content, which showed a significant difference. Moisture content ranged between 76% and 77%, while dry matter content varied from 23% to 24% across the genotypes. Crude protein levels ranged from 6.49% to 7.02%, with the improved variety Desta 01 exhibiting the highest value. Particularly ash content was significantly greater in boiled unpeeled roots (3.77%) compared to their peeled counterparts (2.89%), suggesting better mineral retention. Soluble sugar content varied from 3.94 mg/g DW in LV2 to 5.80 mg/g DW in Desta 01. The result indicates that boiling anchote roots without peeling proved more effective in preserving key nutritional components. Among evaluated genotypes, the Desta 01 genotype is a promising, nutrient‐rich variety with strong potential to contribute to food security and dietary diversification. However, further research is recommended to explore its broader biochemical and pharmaceutical applications.

## Graphical abstract

Anchote, integral to Ethiopian culture, holds significant food, medicinal, social, and economic value. Its superior nutritional values make it a crop of interest for nutrition security. The study found significant impacts of cooking methods on nutritional content, highlighting the importance of considering cooking techniques for preserving essential nutrients.

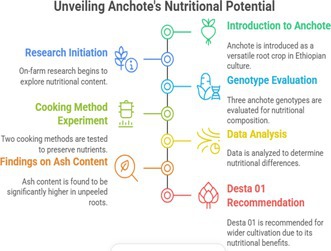

## 1 Introduction

Anchote (*Coccinia abyssinica*), an annual trailing vine crop from the curcubitacae family, is native to Ethiopia. It thrives in the southern and southwestern regions of the country, where its tuberous roots and tender leaves are commonly utilized (Keno 2024). Often grown by farmers for subsistence during lean periods, anchote proves resilient even in less fertile or drought‐prone soils, requiring minimal inputs (Abera et al. 2019). The crop flourishes best at altitudes of 1300–2800 m and with annual rainfall ranging from 762 to 1016 mm (Fekadu et al. 2021).

Anchote is typically propagated by seed, with farmers selecting planting material based on preferred traits such as yield potential, drought tolerance, and cooking quality. Women play a leading role in anchote cultivation, reflecting its cultural significance and gendered agricultural practices in southwestern Ethiopia (Wayessa 2018). The morphology of the root ranges from spherical to conical, a variation largely shaped by environmental conditions (Tessema and Admassu 2021). Harvesting usually takes place four to 5 months after planting, though farmers often leave the tubers in the soil beyond this period. This traditional practice is believed to enhance the crop's medicinal and nutritional properties, further reinforcing its value in both food and health systems (Abera et al. 2019).

With root yields ranging from 8 to 130 t/ha under varying agroecological conditions, anchote is a staple food source and serves medicinal and livestock feed purposes, particularly within the Oromo community (Duresso 2018). Beyond its nutritional contributions, anchote holds deep cultural and social significance, commonly consumed during festive occasions and traditionally used to aid recovery from fractures and to nourish lactating mothers and infants (Wayessa 2018; Parmar et al. 2017).

Anchote, a staple in traditional cuisine, is commonly prepared by boiling before or after peeling, with additional cooking to enhance flavor and digestion. Studies indicate that boiling helps to deactivate enzyme inhibitors and other antinutritional factors, improving nutritional quality. Anchote cooking involves creating a seasoned paste with salt, spices, and butter, enjoyed alone or paired with fermented bread like “injera” (Ayalew et al. 2022; Wayessa 2018; Parmar et al. 2017). Nutritional analyses of anchote have confirmed its superior composition compared to other root crops, including high levels of calcium, protein, and essential trace minerals such as iron, zinc, manganese, and selenium. In particular, white‐fleshed varieties have been reported to meet WHO dietary standards for several key micronutrients (Negassa et al. 2024; Ayalew et al. 2022; Bikila et al. 2022).

In addition to its value as a food crop, anchote has demonstrated wide applicability in food science and material development. Studies have explored the use of anchote starch and extracts in functional food products such as pectin alternatives for jam, stabilizers in fruit juices, and sources of zinc oxide nanoparticles (Bikila et al. 2022; Tiruneh et al. 2021; Safawo et al. 2018). Furthermore, anchote starch has been successfully processed into edible films and cellulose nanocrystals, indicating its potential in sustainable packaging and biomedical materials (Beyan et al. 2022; Kassa et al. 2022). The thermal stability of anchote starch makes it an excellent candidate for thermoplastic starch products, further highlighting the crop's potential as a valuable source of ingredients for food and medicine applications (Abera et al. 2019).

Despite its deep cultural roots and recognized nutritional value, the cultivation of anchote remains localized and largely informal, with minimal integration into national agricultural strategies or global food systems. Although Ethiopia is the center of origin for this crop and harbors substantial genetic diversity, only one improved variety (Desta‐01) has been officially released to date (Bikila et al. 2020). This limited advancement in varietal development underscores the broader neglect of anchote in formal research and development agendas. Given its considerable potential to contribute to nutrition, food security, and even pharmaceutical applications, anchote continues to be an underutilized and insufficiently studied crop both within Ethiopia and globally. Most research to date has focused on general nutritional profiling and industrial applications, with limited attention to varietal differences and how traditional cooking methods affect the retention of nutritional qualities. In practice, anchote is typically boiled either whole or after peeling and slicing, with emerging evidence suggesting that boiling unpeeled roots may better retain nutritional components (Negassa et al. 2024). However, systematic evaluations of how genotype and cooking method interact to influence nutrient composition are lacking.

Therefore, this study was designed to evaluate the proximate composition of three anchote genotypes: two local landraces and the improved variety Desta‐01, under two traditional cooking methods. The aim of this study is to identify anchote genotypes and cooking methods that most effectively preserve key nutrients, thereby generating practical, evidence‐based insights to support the wider adoption of anchote as a nutrient‐rich, culturally significant crop that contributes to dietary diversity, food system resilience, and improved farmer livelihoods in Ethiopia and beyond.

## 2 Materials and Methods

### 2.1 Filed Trial Experiment

**FIGURE 1:**
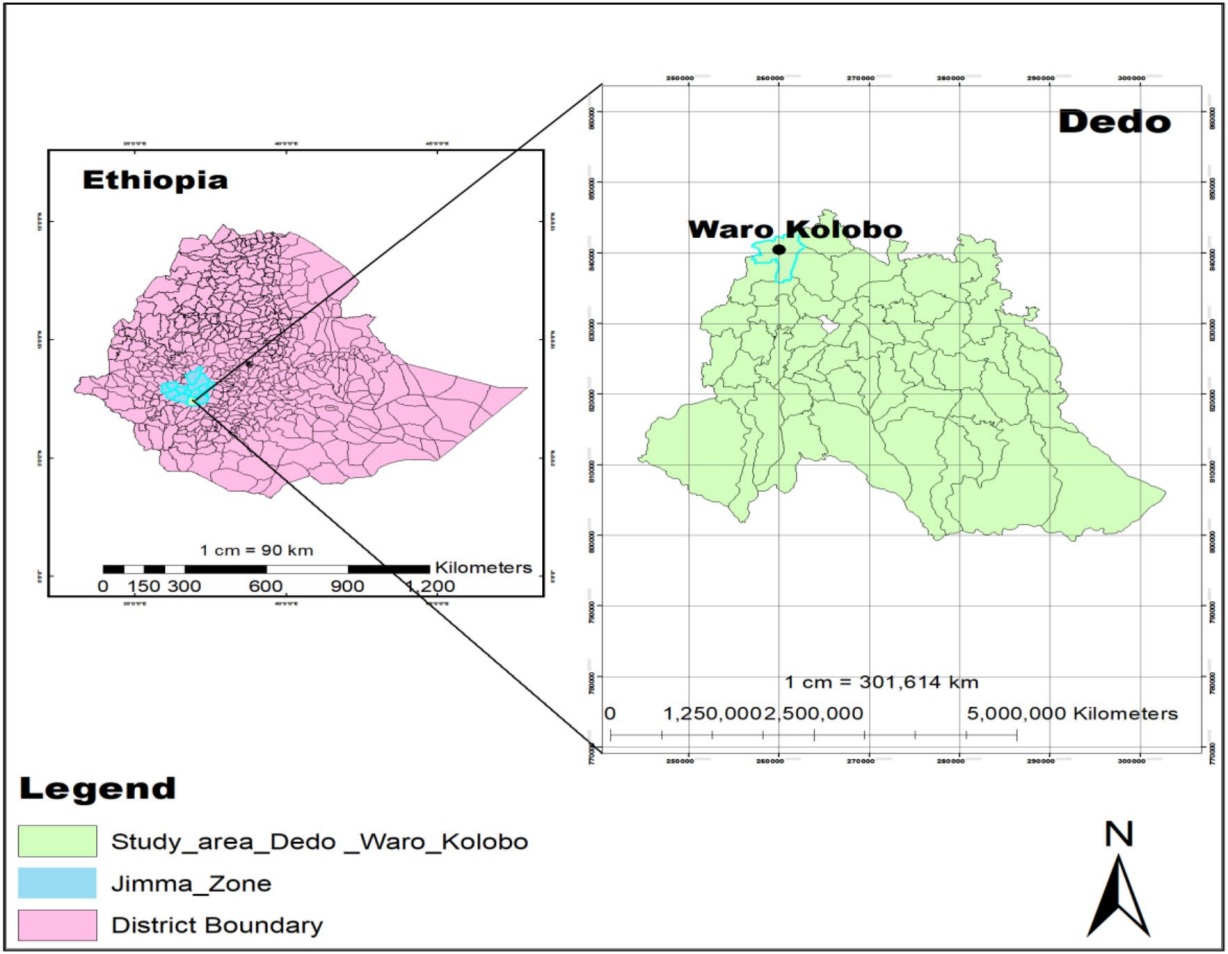
Map of the study Area.

Anchote tubers were cultivated in Waro Kolobo kebele, Dedo district, approximately 20 km south of Jimma town (Figure 1). Production took place in four farmers' fields in an area with an average annual rainfall of 1710.3 mm and temperatures ranging from 12.2°C to 25.6°C. The region also grows other root and tuber crops, along with various horticultural crops, on a predominantly Nitisol soil type.

**FIGURE 2:**
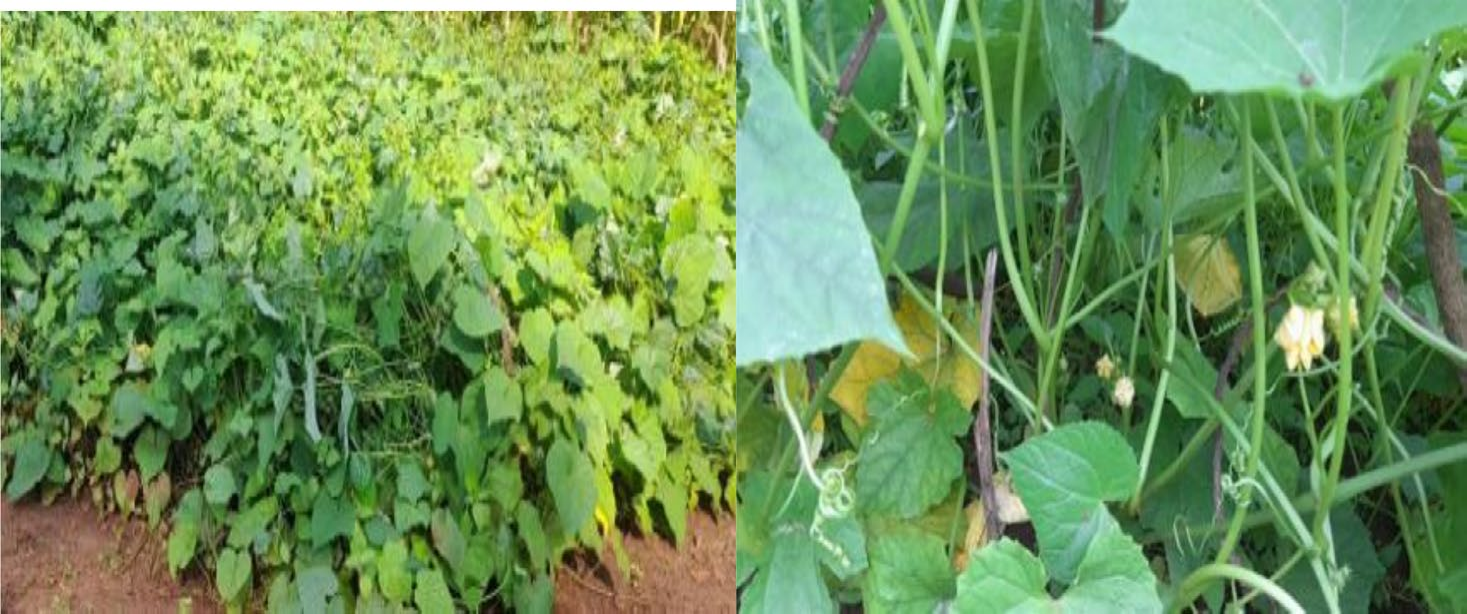
Field layout during the anchote on‐farm trial in Waro Kolobo.

The farm trial involved three anchote genotypes: two local landraces (LV1‐1 and LV‐2) and an improved variety named Desta‐01, cultivated in plots of 5 m × 2 m across four blocks tended by individual farmers (Figure 2). Standard agronomic practices, such as fertilizer application, weeding, and harvesting, were carried out following recommendations from the Debrezeit Agricultural Research Center (Negassa et al. 2024). Planting was done on June 4, 2022, with seeds spaced 60 cm apart in rows and 20 cm between plants. After 5 months, harvesting was done in the first week of November 2022, coinciding with leaf withering and root maturation.

### 2.2 Sample Preparation and Treatment Application in the Laboratory

**FIGURE 3:**
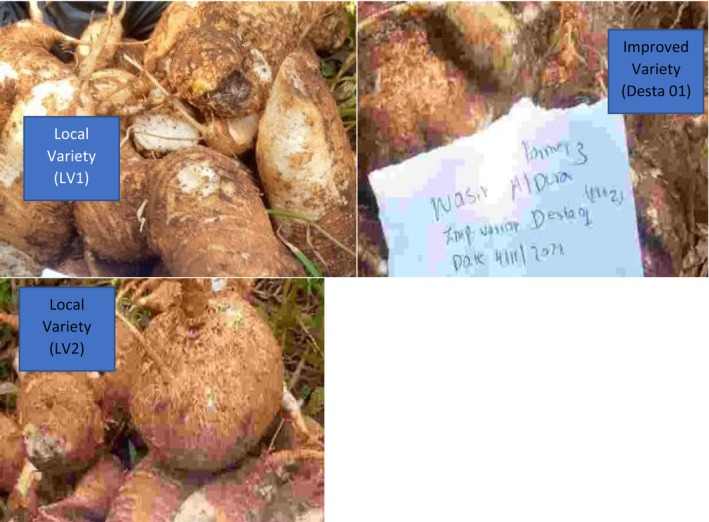
Root of variety used for proximate and soluble sugar analysis.

The roots from 15 plants of the three genotypes (Figure 3) from each of the four farmers' fields were harvested and transported to Jimma in labeled poly bags. In Jimma, the roots were cleaned, rootlets were cut according to genotypes and replications and then boiled in labeled pans. Some roots were peeled, sliced, and boiled, while others were boiled without peeling. After boiling, the roots were sliced, dried in an oven at 90°C for 72 h, ground, sieved, packed in labeled bags, and stored in a freezer at −20°C.

### 2.3 Data Collection

#### 2.3.1 Moisture Content and Dry Matter of the Roots

The fresh weight of the boiled peeled and sliced Anchote root samples and the boiled unpeeled whole root samples of each of the three genotypes from each of the four replications was weighed and oven dried at 92°C for 72 h until a constant mass was achieved. The dry matter was calculated by weighing the dried samples, dividing by the fresh weight, and multiplying by 100. The moisture content of the samples was calculated by deducting the percentage of the dry matter calculated against the fresh weight.

#### 2.3.2 Proximate Analysis

The samples of the boiled peeled and sliced storage root and the boiled unpeeled whole storage root of each of the three genotypes of anchote from each replication were weighed in 10 g and transferred into the labeled centrifuge tubes. The samples were sent to the Nova Scotia Agricultural Laboratory Services for the proximate analyses. The crude protein was determined by combustion method using Leco CN828 CN Determinator (AOAC‐990.03, Association of Official Analytical Chemists [AOAC] 2011). Ash was determined by the AOAC combustion method (AOAC 942.05). The dry matter content and moisture content of anchote flour were determined by loss on drying or gravimetric method (AOAC‐935.29). The acid detergent fiber (ADF) and neutral detergent fiber (NDF) were determined using an Ankom fiber analyzer.

#### 2.3.3 Soluble Sugars

Soluble sugars were extracted using a modified method by Blunden and Wilson (1985). Approximately 0.2 g of powdered anchote root sample was treated with 90% ethanol, followed by centrifugation and spectrometric analysis using phenol‐sulfuric acid. Absorbance was measured at 490 nm, and sugar content was quantified using a glucose standard curve. Results were expressed as percentage glucose per dry weight basis.

## 3 Results

### 3.1 Proximate Analysis

#### 3.1.1 Moisture Content and Dry Matter of the Roots

The analysis of variance showed that the moisture content and dry matter content of anchote roots were not significantly affected by genotype (*p* > 0.05) or by the interaction between genotype and cooking method (*p* > 0.05) (Tables 1, 2, 3).

**TABLE 1:** The mean values of the proximate composition of Anchote (*Coccinia abyssinica*) flour for the cooking methods.

Genotype	Moisture content of roots (%)	Dry matter of content of the roots (%)	Soluble sugar in mg/g of DW
Land race 1	76^a^	24^a^	4.70^a^
Land race 2	77^a^	23^a^	3.94^a^
Improved variety Desta 01	76^a^	24^a^	5.80^a^
SD	0.589	0.589	0.65
CV (%)	0.77	2.52	13.5
*p*	0.73	0.730	0.55

*Note:* Within each column, means sharing the same letter are not significantly different at *α* = 0.05 level of significance.

**FIGURE 4:**
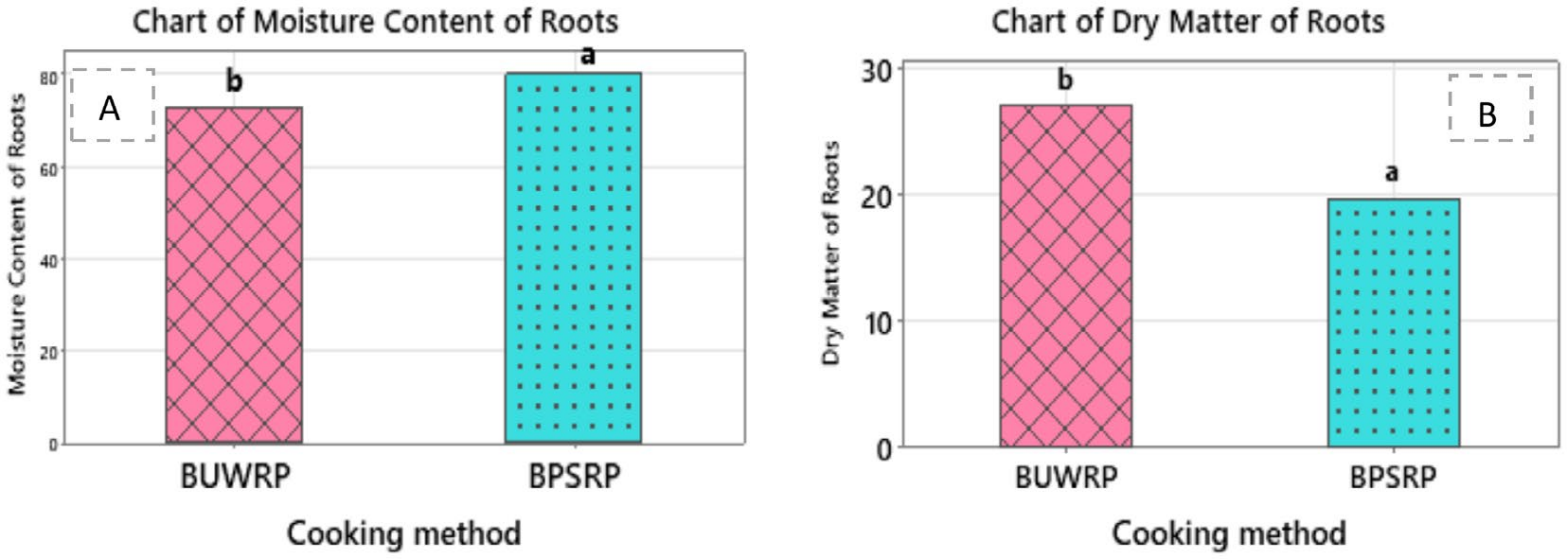
Mean values of the moisture content (%) (A) and dry matter of roots (%) (B) in response to the cooking method. BUWRP, boiled unpeeled whole roots, BPSRP, boiled peeled and sliced roots.

On a fresh weight basis, the genotypes Desta 01 and LV1 recorded moisture contents of 76%, while their dry matter contents were 24%. The landrace LV2 had a slightly higher moisture content (77%) and a lower dry matter content (23%). Comparing the cooking methods, boiled peeled and sliced roots had a higher moisture content (80%) and lower dry matter content (20%) than boiled unpeeled whole roots, which had 73% moisture and 27% dry matter, respectively (Table 1). This indicates that boiling roots without peeling helps retain more dry matter, likely due to reduced water absorption during cooking. The mean differences between cooking methods are further illustrated in Figure 4.

Similarly, for anchote flour, moisture and dry matter contents were not significantly affected by genotype, cooking method, or their interaction (*p* > 0.05) (Tables 1, 2, 3). Moisture contents of the flour prepared from LV2, Desta 01, and LV1 were 6.41%, 6.17%, and 6.19%, respectively, while the corresponding dry matter contents were 93.59%, 93.83%, and 93.81% (Table 2). Flour derived from boiled peeled and sliced roots had a moisture content of 6.35% and a dry matter content of 93.64%, while that of boiled unpeeled whole roots had a moisture content of 6.16% and a dry matter content of 93.83% (Table 1). These values fall within the acceptable range for flour quality and stability. The dry matter values obtained in this study were higher than those reported by Gemede and Fekadu (2014), who found dry matter contents of 18.26% and 23.27% in peeled and unpeeled boiled anchote roots, respectively. The difference may be attributed to variations in genotype, harvest maturity, and cooking or drying conditions.

**TABLE 2:** The mean values of moisture content and dry matter content of anchote roots for the three genotypes.

Processing method	Dry matter of anchote flour (%)	MC of anchote flour (%)	Crude protein (%)	ADF (%)	NDF (%)	Ash (%)
BPSTP	93.64^a^	6.35^a^	6.67^a^	8.32^a^	12.61^a^	2.89^b^
BUWTP	93.83^a^	6.16^a^	7.00^a^	7.91^a^	11.94^a^	3.77^a^
SD	0.134	0.1338	0.235	0.291	0.477	0.625
CV (%)	0.14	2.14	3.43	3.59	3.89	18.76

*Note:* Within each column, means sharing the same letter are not significantly different at *α* = 0.05 level of significance.

#### 3.1.2 Crude Protein

The analysis of variance indicated that crude protein content was not significantly affected by genotype, cooking method, or their interaction (*p* > 0.05) (Table 3). Among the genotypes, LV1, LV2, and Desta‐01 had mean crude protein contents of 7.02%, 6.49%, and 7.00%, respectively (Table 2). Regarding cooking methods, the boiled peeled and sliced roots contained 6.67% crude protein, while the boiled unpeeled whole roots contained a slightly higher value of 7.00% (Table 1).

**TABLE 3:** The mean values of the proximate compositions of the interaction effect of the genotypes and the cooking methods.

Genotype × Method	Dry matter of anchote flour (%)	Moisture content of anchote flour (%)	Crude protein%	ADF%	NDF%	Ash (%)	Soluble sugar in mg/g of DW
LV1 BUWTP	94.09^a^	5.90^a^	6.89^a^	7.64^a^	10.52^a^	3.53^abc^	5.69^a^
LV2BUWTP	93.78^a^	6.22^a^	7.48^a^	8.14^a^	12.82^a^	3.67^ab^	4.21^a^
IVD01BUWTP	93.64^a^	6.36^a^	6.65^a^	7.95^a^	12.48^a^	4.12^a^	5.92^a^
LV1BPSTP	93.53^a^	6.47^a^	7.13^a^	7.65^a^	12.28^a^	2.71^d^	3.71^a^
LV2BPSTP	93.4^a^	6.6^a^	6.56^a^	8.64^a^	11.58^a^	2.86^cd^	3.68^a^
IVD01 BPSTP	94.02^a^	5.98^a^	6.34^a^	8.67^a^	13.97^a^	3.1^bcd^	5.68^a^
SD	0.276	0.276	0.414	0.457	1.165	0.537	1.062
CV (%)	0.29	4.4	6.05	5.64	9.49	16.11	22.05

*Note:* Within each column, means sharing the same letter are not significantly different at *α* = 0.05 level of significance.

These protein levels were higher than those reported by Gemede and Fekadu (2014), who found values ranging from 2.67% to 3.14%, and by Bikila et al. (2020), who reported a range of 3.25%–3.42%. The observed differences may be attributed to variations in genotype, harvest maturity, and differences in cooking or drying conditions. The relatively high protein content found in this study reinforces the nutritional value of anchote as a promising root crop, particularly for communities that rely on tubers as staple foods.

#### 3.1.3 Acid Detergent Fiber and Neutral Detergent Fiber

**FIGURE 5:**
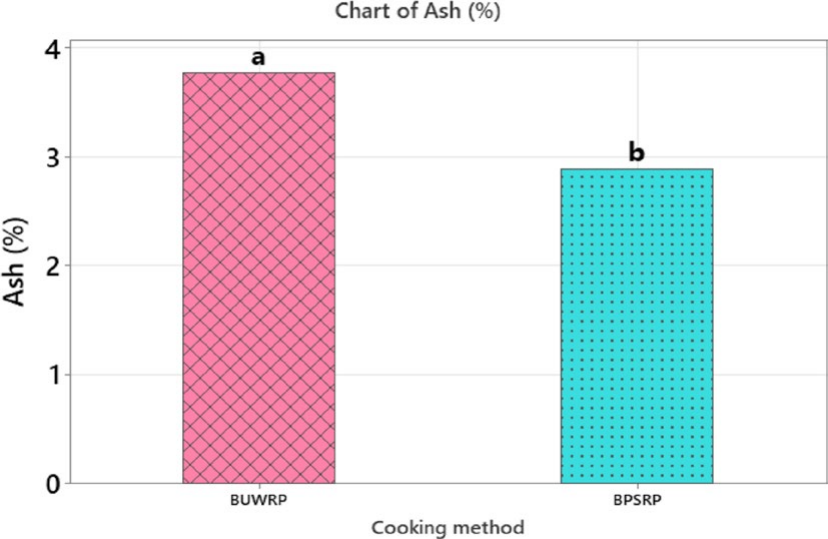
Comparison of the mean values of ash in response to the cooking method at *α* = 0.05. BUWRP, boiled unpeeled whole roots; BPSRP, boiled peeled and sliced roots.

The analysis of variance showed that Acid Detergent Fiber (ADF) content was not significantly affected by genotype, cooking method, or their interaction (*p* > 0.05) (Tables 1, 2, 3). The mean ADF values were 8.39% for LV2, 8.31% for LV1, and 7.65% for Desta‐01 (Table 2). With regard to cooking methods, the boiled peeled and sliced roots contained 8.32% ADF, whereas the unpeeled whole roots had a slightly lower value of 7.9% (Figure 5).

Neutral Detergent Fiber (NDF) content also did not show any significant differences due to genotype, cooking method, or their interaction (*p* > 0.05) (Tables 1, 2, 3). The mean NDF values were 13.22% for Desta‐01, 12.20% for LV2, and 11.40% for LV1 (Table 3). Boiled peeled and sliced roots had an NDF content of 12.61%, compared to 11.94% in the unpeeled whole roots.

The ADF and NDF values reported in this study were lower than those reported by Bikila et al. (2020), who found NDF and ADF levels of 23.6% and 11.6%, respectively, and by Banti et al. (2025), who reported even higher NDF values ranging from 33.7% to 58.3%. The lower fiber values in this study may be attributed to the cooking and drying processes, which likely reduced fiber concentration through heat‐induced structural breakdown or leaching.

#### 3.1.4 Ash Content

Ash content was not significantly affected by genotype (*p* > 0.05) or the interaction between genotype and cooking method (*p* > 0.05) (Tables 2 and 3). However, the cooking method had a significant effect on ash content (*p* < 0.05) (Table 1). Among the genotypes, the mean ash contents were 3.61% for Desta 01, 3.26% for LV2, and 3.12% for LV1. With respect to cooking methods, boiled unpeeled whole roots had a higher ash content (3.77%) compared to peeled and sliced roots (2.89%), suggesting that boiling without peeling helps retain more minerals. This difference may be attributed to mineral leaching during boiling after peeling. A comparison of ash content by cooking method is presented in Figure 5.

**FIGURE 6:**
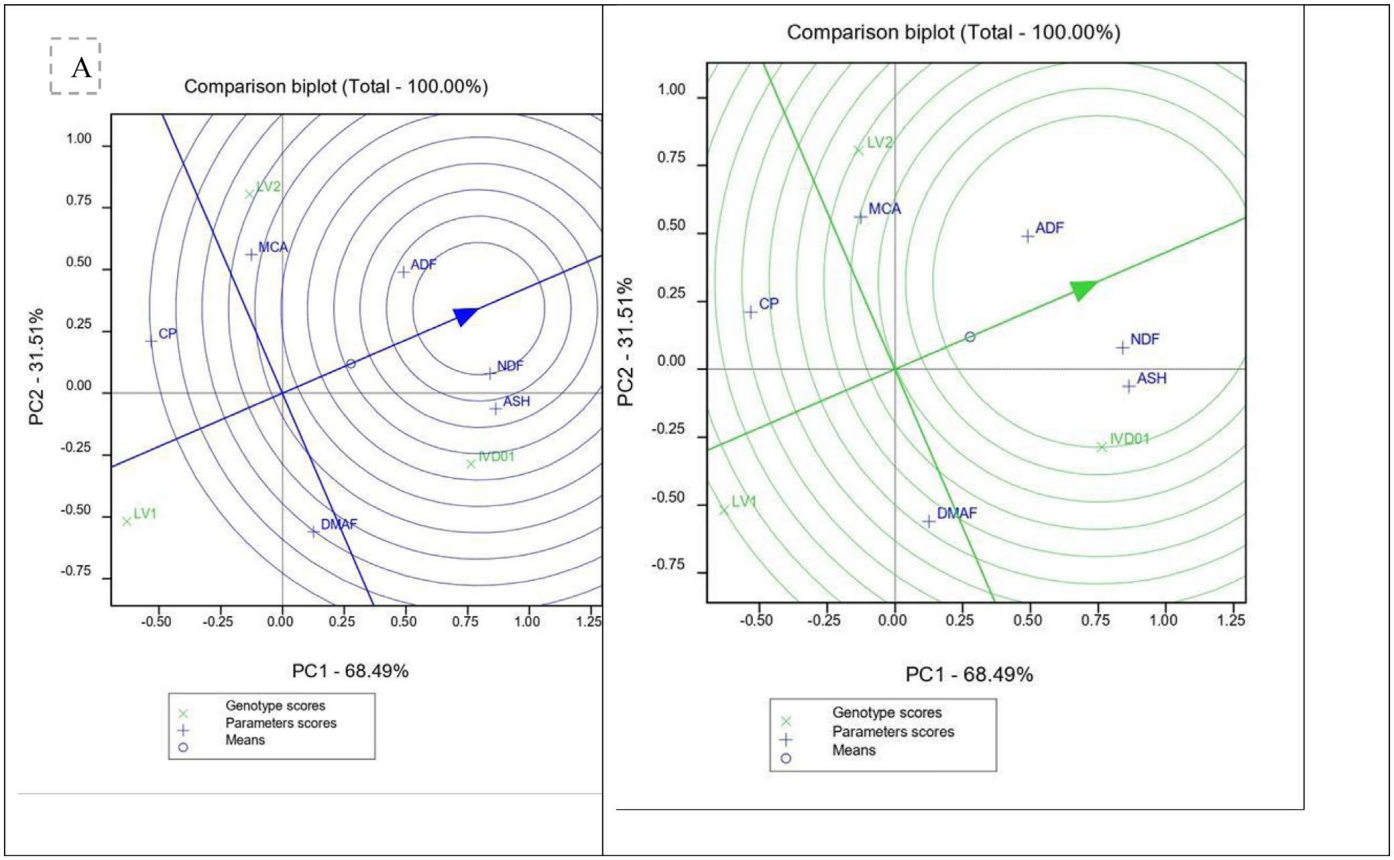
The PCA comparison bi‐plot of the proximate compositions with the genotypes (A is for the parameters projection and the Variety Association while B is for variety projection and association with the parameters).

**FIGURE 7:**
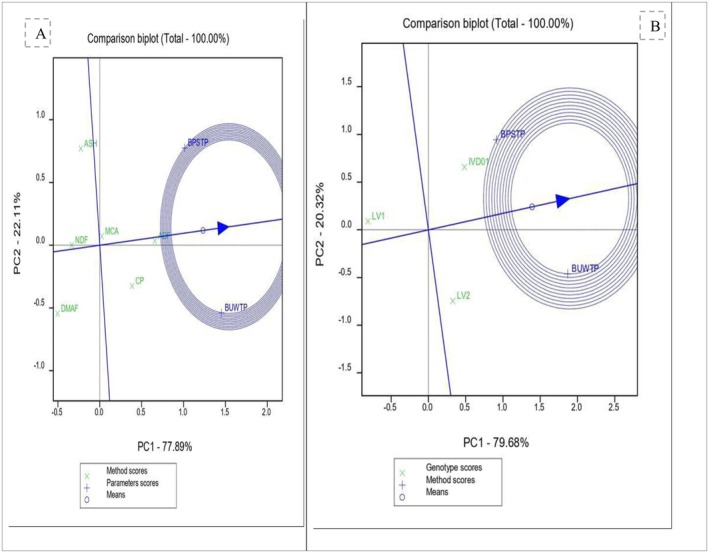
The PCA comparison bi‐plot of the genotypes and the cooking methods in terms of proximate composition (A is the projection for proximate compositions to the cooking method and B shows the association of the genotypes and cooking methods).

Principal Component Analysis (PCA) results showed that more than 99% of the variation in proximate composition among genotypes and cooking methods was captured by the first two components (Figures 6 and 7). Among the genotypes, Desta 01 exhibited the most favorable proximate composition, followed by LV2, while LV1 had the lowest. Regarding cooking methods, boiling without peeling consistently preserved higher concentrations of proximate nutrients than boiling after peeling and slicing.

### 3.2 Soluble Sugar

Soluble sugar content was not significantly affected by genotype, cooking method, or their interaction (*p* > 0.05 in all cases) (Tables 1, 2, 3). Among the genotypes, Desta 01 had the highest mean soluble sugar content at 5.80 mg/g dry weight (DW), followed by LV1 at 4.70 mg/g DW and LV2 at 3.94 mg/g DW (Table 3). In terms of cooking method, boiled unpeeled whole roots contained more soluble sugar (5.28 mg/g DW) compared to boiled peeled and sliced roots (4.36 mg/g DW) (Table 3), indicating that cooking without peeling better preserves soluble carbohydrates.

Soluble sugars are important carbohydrates that influence the organoleptic properties such as taste and sweetness of plant‐based foods. The presence of measurable soluble sugars in anchote agrees with the findings of Bikila et al. (2022). However, the soluble sugar contents observed in this study were lower than those reported by Noman et al. (2007) for potato (4.8%, or 48 mg/g DW) and sweet potato (5.6%, or 56 mg/g DW). The lower sugar concentration in anchote may be attributed to its naturally less sweet flavor and the boiling process, which can reduce sugar content through leaching and thermal degradation. Nonetheless, the relatively low sugar content of anchote makes it a suitable dietary option for individuals managing blood sugar levels, including diabetic patients, and supports its use in healthy nutrition programs.

## 4 Discussion

The findings on moisture and dry matter content of the anchote genotypes (Table 2) and cooking methods (Table 1) were inconsistent with the results of Gemede and Fekadu (2014), who reported a moisture content of 81.74 g/100 g and a dry matter content of 18.26 g/100 g for peeled and boiled roots. For unpeeled roots, they reported a moisture content of 76.73 g/100 g and a dry matter content of 23.27 g/100 g. The higher moisture and lower dry matter content in peeled roots may be due to increased water absorption by the exposed starch and fiber during boiling. In contrast, unpeeled roots are better protected by the outer skin, which likely limits moisture uptake and nutrient loss. Other authors have reported moisture and dry matter ranges for raw anchote roots between 70.44%–80.17% and 19.83%–29.56%, respectively (Ayalew et al. 2022; Parmar et al. 2017). These differences may stem from variations in genotype, maturity stage at harvest, drying methods, and temperature. Comparable moisture and dry matter values have also been reported for sweet potato (77.28% and 22.72%, respectively), further supporting the suitability of anchote as both a fresh food crop and a raw material for processing (Kuyu et al. 2018; Parmar et al. 2017). Boiling whole roots without peeling is preferable for retaining higher dry matter content and limiting moisture gain.

For anchote flour, the moisture and dry matter contents (Table 2, Figure 5) were lower than those reported by Bikila et al. (2020), who found moisture contents of 9.8–10.1 g/100 g for boiled, peeled anchote roots. The differences could be due to variation in cooking and drying temperatures. However, the moisture content observed in this study (6.16%–6.41%) aligns with the recommended threshold of 6.71% for cassava flour, ensuring good quality, extended shelf life, and microbial stability (Chukwu and Abdullahi 2015).

The crude protein content of the anchote genotypes (Table 1) and cooking methods (Table 2) ranged from 6.49% to 7.02%, which was higher than those reported by Gemede and Fekadu (2014) (2.67–3.14 g/100 g) and by Bikila et al. (2020) (3.25–3.42 g/100 g). This discrepancy may be attributed to differences in genotype, harvest maturity, and processing conditions. Heat‐induced protein denaturation may also play a role in the variation. The values in this study were also higher than those for raw anchote reported by Parmar et al. (2017) at 2.77 g/100 g, though still lower than the 16.4% reported by Ayana (2019). The observed decrease in protein content from raw to boiled roots is consistent with earlier findings by Gemede and Fekadu (2014) and Bikila et al. (2020).

Compared to other root and tuber crops, anchote displayed superior protein content. Sweet potato contains approximately 0.57% protein (Kuyu et al. 2025), cassava 0.3%–3.5% (Salvador et al. 2014), and yam species 1%–3% (Shewry 2003). Taro was reported at 2% by Shewry and up to 9% by Banti et al. (2025). Therefore, the protein content of anchote (6.49%–7.02%) was considerably higher than that of most root crops, making it a reliable protein‐rich food source, particularly in communities that rely on tuber crops as dietary staples.

In addition to human consumption, anchote is widely used by farmers in West Wollega for livestock fattening. Its leaves and vines are known to have high protein and fiber contents, particularly ADF, NDF, ADL, and ash. The ADF and NDF values observed in this study (Table 3, Figure 5) were lower than those reported for raw anchote: 11.6% ADF and 23.6% NDF (Bikila et al. 2020), and 33.7%–58.3% NDF (Banti et al. 2025). The lower fiber values could be attributed to cooking and drying temperatures that degrade fibrous components. Nevertheless, thermal processing is known to reduce antinutritional factors, enhancing the bioavailability of nutrients (Gemede and Fekadu 2014). Further research should explore digestibility and nutrient utilization in animals to support the wider use of anchote as a feed resource at commercial scales.

The ash content of the genotypes (Table 2) was consistent with values reported by Bikila et al. (2020), ranging from 2.96 to 3.25 g/100 g. However, the boiled peeled and sliced roots showed a slightly lower ash content (2.89%), whereas the boiled unpeeled whole roots showed a higher value (3.77%). These findings differ from Gemede and Fekadu (2014), who reported 1.33 g/100 g for peeled and 1.99 g/100 g for unpeeled boiled roots. The variation may result from differences in genotype, soil conditions, maturity stage, and processing methods. The observed increase in ash content in unpeeled roots suggests that mineral retention is better preserved when the skin remains intact during cooking, while peeling may promote leaching of minerals.

The ash content of anchote also exceeded that of cassava, which ranges from 0.4 to 1.7 g/100 g (Salvador et al. 2014). Since ash content is directly correlated with the presence of inorganic minerals, samples with higher ash values are expected to contain higher concentrations of essential nutrients (Negassa et al. 2024; Bikila et al. 2020). This gives anchote a comparative advantage in promoting metabolic processes and supporting human growth and development. Hence, anchote stands out as a rich source of minerals, regardless of genotypic differences.

## 5 Conclusion

This study highlights the significant nutritional potential of anchote, particularly the improved genotype Desta 01, which demonstrated superior levels of protein and ash content compared to local landraces. The findings underscore the impact of cooking methods on nutrient retention, with boiling unpeeled roots emerging as the most effective technique for preserving nutritional quality. Anchote's comparatively higher protein and mineral content, alongside its low moisture content, positions it as a promising crop for enhancing food and nutrition security in Ethiopia and similar contexts. Additionally, its potential use as animal feed offers added value, supporting broader agricultural sustainability. In conclusion, promoting Desta 01 for wider cultivation and advocating for unpeeled boiling as a standard processing method are strongly recommended. Further research into the crop's biochemical properties and its applications in both human and livestock nutrition will help to unlock its full potential.

## Author Contributions

**Bogalech Negassa:** resources (equal), validation (equal), visualization (equal), writing – original draft (lead), writing – review and editing (equal). **Amsalu Nebiyu:** resources (equal), software (equal), supervision (equal), validation (equal), visualization (equal), writing – original draft (equal), writing – review and editing (equal). **Weyessa Garedew:** resources (equal), supervision (equal), visualization (equal), writing – original draft (equal), writing – review and editing (equal). **Lord Abbey:** supervision (equal), validation (equal), visualization (equal), writing – original draft (equal), writing – review and editing (equal). **Raphael Ofoe:** supervision (equal), validation (equal), visualization (equal), writing – original draft (equal). **Tessema Astatkie:** validation (equal), visualization (equal), writing – review and editing (equal). **Chala Gowe Kuyu:** validation (equal), visualization (equal), writing – original draft (equal), writing – review and editing (lead).

## Funding

This work was supported by the College of Agriculture and Veterinary Medicine, Jimma University, NA. The field work of this paper has received funding from the European Union's Horizon 2020 Research and Innovation Programme (Grant Agreement Number 862848).

## Ethics Statement

The study adhered to the ethical principles outlined in Jimma University's approved rules and regulations for conducting research. The Research and Ethical Review Board (RERB) of the College of Agriculture and Veterinary Medicine of Jimma University comprehensively reviewed the project and approved its implementation.

## Consent

The authors have nothing to report.

## Conflicts of Interest

The authors declare no conflicts of interest.

## Data Availability

Data are available upon request from the corresponding author.
